# Obesity Impact on the Attentional Cost for Controlling Posture

**DOI:** 10.1371/journal.pone.0014387

**Published:** 2010-12-20

**Authors:** Jean-Baptiste Mignardot, Isabelle Olivier, Emmanuel Promayon, Vincent Nougier

**Affiliations:** 1 Laboratoire TIMC-IMAG, Team SPM (Santé – Plasticité – Motricité), UMR UJF-CNRS 5525, La Tronche, France; 2 Laboratoire TIMC-IMAG, Team GMCAO (Geste Médicaux Chirurgicaux Assistés par Ordinateur), UMR UJF-CNRS 5525, La Tronche, France; Institute of Preventive Medicine, Denmark

## Abstract

**Background:**

This study investigated the effects of obesity on attentional resources allocated to postural control in seating and unipedal standing.

**Methods:**

Ten non obese adults (BMI = 22.4±1.3, age = 42.4±15.1) and 10 obese adult patients (BMI = 35.2±2.8, age = 46.2±19.6) maintained postural stability on a force platform in two postural tasks (seated and unipedal). The two postural tasks were performed (1) alone and (2) in a dual-task paradigm in combination with an auditory reaction time task (RT). Performing the RT task together with the postural one was supposed to require some attentional resources that allowed estimating the attentional cost of postural control. 4 trials were performed in each condition for a total of 16 trials.

**Findings:**

(1) Whereas seated non obese and obese patients exhibited similar centre of foot pressure oscillations (CoP), in the unipedal stance only obese patients strongly increased their CoP sway in comparison to controls. (2) Whatever the postural task, the additional RT task did not affect postural stability. (3) Seated, RT did not differ between the two groups. (4) RT strongly increased between the two postural conditions in the obese patients only, suggesting that body schema and the use of internal models was altered with obesity.

**Interpretation:**

Obese patients needed more attentional resources to control postural stability during unipedal stance than non obese participants. This was not the case in a more simple posture such as seating. To reduce the risk of fall as indicated by the critical values of CoP displacement, obese patients must dedicate a strong large part of their attentional resources to postural control, to the detriment of non-postural events. Obese patients were not able to easily perform multitasking as healthy adults do, reflecting weakened psycho-motor abilities.

## Introduction

Many studies investigated postural control in different pathological or aging populations in order to optimize programs for prevention and/or rehabilitation of postural disorders. However, regarding obesity, the literature studying the impact of this disease on postural control remains scarce. Obesity is a major scourge in developed countries in which its prevalence increased severely in the past two decades [1]–[3].

Besides its negative impact on a large number of physiological functions, it facilitates the development of associated pathologies such as hypertension, diabetes, hyperlipidemia, coronary heart disease, heart failure, respiratory failure, cholelithiasis, osteoarticular disease, and several cancers [4], [5]. In addition, some recent studies highlighted the effects of this disease on the musculoskeletal system [6], [7], or motor skills and balance [8]–[11]. These studies generally reported in obese patients an increase of the centre of foot pressure (CoP) oscillations during maintained bipedal stance and a slower walking speed.

The ability to control postural balance and an erect posture requires complex sensory-motor and cognitive processes [12]. However, to our knowledge no study has focused on quantifying the attentional cost of maintaining a given posture in obese patients. How attentional resources are allocated is however an important indication to assess the degree of postural control. For example, adding to the control of posture a second cognitive task such as a verbal or motor reaction time to auditory or visual stimuli (RT) allows quantifying the allocation of attention necessary for the control of this posture. For example, whereas in adult healthy subjects small or no attentional costs are generally observed for controlling posture, [13] have shown that the attentional cost involved in postural control increases with ageing. Indeed, many studies showed, in a dual-task condition, an increased RT in elderly subjects as compared to young ones [11]–[17] or among elderly people between a single reference posture (e.g., sitting) and a more complex erected posture [16], [18], [19] or between the dual and single phases of support during walking [20]. According to Lajoie et al. (1993) [20], a higher attentional demand is required when sensory information is altered or reduced, or when complexity of the postural task increases. In a dual-task condition, an increased RT can thus be considered as a marker of an increased postural task difficulty [14], [15], [19]. Indeed, some authors have even established a positive correlation between RT increase and the increased risk of falls among elderly [15], [21]. On the basis of these various studies in elderly people, similar results could be expected in obese patients.

Whereas for normal aging or for some pathology such as spinal cord injury [22] or stroke patients [12] many studies investigated the allocation of attentional resources during postural or motor tasks, this question does not seem to have been investigated for the obese population. This is why we aimed at evaluating the impact of obesity on the attentional cost required for maintaining two different postures (seating and unipedal standing). Contradictions in the literature regarding bipedal stability in obese patients suggested that this posture is not complex enough to reach clear conclusions [23], [24]. In day life situations, equilibrium disturbances generally occur in a more complex biomechanical and cognitive context [25], especially during postural transitions (from bipedal to unipedal posture and vice-versa) as for example during gait in which the swinging phase of one foot requires a fine control of the projection of the centre of gravity in the reduced base of support of the supporting foot. Unipedal posture seems to be a good compromise: Given the significant reduction of the bearing surface, it increases the difficulty of maintaining a stable posture and allows a parallel evaluation of CoP oscillations.

The goal of the present study was to quantify postural stability in non obese and obese patients and the attentional cost required to maintain balance in more or less complex postural tasks. In a dual-task paradigm, combination of centre of foot pressure (CoP) analysis and RT data in non obese and obese patients should allow evaluating patients' postural control in relation to the risk of falling. Estimating the contribution of the attentional resources allocated to the central processes involved in postural control should allow a better understanding of the origin of postural disturbances in obese patients in order to optimise the therapeutic intervention for stopping the obesity deconditioning process [26].

## Methods

### Ethics Statement

“Comité de Protection des Personnes”, zone Sud Est V, France, Joseph Fourier University and Clinical Trials (NCT01106105) has specially approved this study.

### Participants

Ten healthy non-obese adults (five women and five men; mean age = 42.4±15.1 and mean Body Mass Index (BMI) = 22.4±1.3) and ten obese adults (five women and five men; mean age = 46.2±19.6 and mean BMI = 35.2±2.8; *F*(1,18) = 0.3, p>0.05, and 176.3, p<0.001, for control vs. obese age and BMI, respectively) voluntarily took part in this investigation.

All participants underwent a complete medical examination and only individuals free from known muscular, neurological or cardiovascular deficits took part in the study. Only those individuals taking part in recreational, non competitive, physical activities at a frequency of no more than twice a week were admitted to the study. Written informed consent was obtained, and all experimental procedures conformed to the standards set by the Declaration of Helsinki and Huriet law, were approved by the local ethics committee on human research, and were supported by the French research ministery.

### Postural task

Participants were asked to stay as immobile as possible for 20 sec on a force platform. They fixated a white cross (20×20 cm) located 3 m away from the force platform, at eyes level. Participants were instructed to keep their body straight and their arms loosely hanging by their sides. Two postural conditions were investigated. In the first condition (Seated), participants seated on the force platform so that 2/3 of the proximal thighs length touched the force platform with the arms crossed on the top of the tights. In the second postural condition (Unipedal), participants stood on the force platform with their preferred foot; the other foot was lifted so that it had no contact with the support surface.

### Reaction time task

An additional auditory reaction time task (RT) was performed on some trials. Participants were instructed to respond as fast as possible to the auditory stimulus by a finger pressure exerted on a contactor. Over a 20 sec trial, 10 auditory stimuli (sound frequency was 1000 Hz) were randomly presented through a loudspeaker located 1 m away from the ear canal. In order to prevent anticipation, two successive stimuli were separated by a random interval of 0.8 to 2 sec by steps of 0.2 sec. In the dual task context, no instruction was given regarding the priority dedicated to the postural and RT tasks.

### Procedure

Participant performed two experimental conditions. In the single task condition, participants performed the postural task alone. In the dual–task condition, participants performed the postural task together with the RT task. Four trials for each posture and each single and dual-task condition were performed for a total of 16 trials. The order of presentation of the trials was randomized across participants. A one min rest separated blocks of four successive trials

### Data analysis

Signals from the force platform (AMTI model OR 6–7) were sampled at 100 Hz (12-bit A/D conversion) and low pass filtered with a second-order Butterworth (10 Hz). Displacement of the CoP was then assessed by computation of the three orthogonal components of the ground reaction forces and their associated torque. Two dependent variables were used to describe participants' postural behaviour. The range of CoP displacements indicated the maximal excursion of the CoP in any direction. It is a global measure that allows estimating overall postural performance (i.e., stability). The speed of CoP displacements was the sum of the displacement scalars (i.e., the cumulated distance over the sampling period) divided by the sampling time. This measure has been suggested to represent the amount of activity required to maintain stability, providing a more functional approach of postural control [27]. To nullify the effect of the anthropometric factors on the inverse pendulum model of postural control [28], all postural data were normalised with respect to body height. Analysis of postural data was performed on trials without a consecutive fall. However, for the obese patients, maintaining an unipedal posture for more than 5 sec was a real challenge. Considering these functional difficulties, we decided to take into consideration all trials exceeding 10 sec of duration without equilibrium loss. It must be emphasised that decreasing the collection time from 20 to 10 sec did not affect the data analysis since CoP range was a displacement data time independent and CoP speed was normalised with respect to the duration of a trial.

### Statistical Analysis

All data are reported as mean values ± standard deviation. Two-way repeated-measures analyses of variance (ANOVAs) were applied to the dependent variables, depending on the conditions, to investigate differences between groups and / or conditions. *Post hoc* analyses were assessed using the HSD Tukey test whenever necessary. The software package Statistica (Statsoft, version 6.0, Statistica, Tulsa, OK) was used for all analyses.

## Results

In the unipedal postural condition for the obese patients, 7.3 trials±1.8 in average were necessary for validating 4 good trials, which meant a rate of success of 59%±20. In the same postural condition, control participants exhibited a 100% rate of success.

Analysis of CoP showed significant main effects of group [*F*(1,18) = 16.44 and 22.45, ps<.001, for speed and range, respectively] and posture [*F*(3,54) = 99.84 and 249.52, ps<.001, for speed and range, respectively]. As illustrated in figure 2, results also showed a significant interaction of group×posture [*F*(3,54) = 15.8 and 10.9, ps<.001, for speed and range, respectively]. The decomposition of the interaction into its simple main effects showed that in the seated position whatever the simple or dual-task condition, control and obese CoP displacement was similar **(** **figure 2** **)**. In the unipedal posture, however, CoP speed and range increased much more in obese patients than in control participants whatever the single or dual-task condition (obese CoP speed increased about 120.93% and 99.32% (**figure 2a**) and range increased about 46.12% and 60.93% (**figure 2b**) in the single and dual-task conditions, respectively).

Analysis of RT, showed main effects of group [*F*(1,18) = 12.18, p<.01] and posture [*F*(1,18) = 10.59, p<.01], and a significant interaction of group×posture [*F*(1,18) = 4.97, p<0.05].

The decomposition of the interaction into its simple main effects showed that for the control participants, RT remained similar whatever the postural complexity whereas it severely increased for the obese patients in the unipedal stance.

## Discussion

Many studies assessing attentional cost while maintaining static or dynamic postures were used as a base of reference for postural control [12], [19], [20], [29], [30]. Generally, the postural context is a simple motor task in which the attentional cost required to maintain equilibrium is rather low as compared to many day life sensori-motor situations such as walking, grasping an object on the ground, or moving from a sitting to standing position. When seated in the present experiment, no effect of group on CoP sway or RT were observed (figures 2 and 3 and table 1). This postural condition may be considered as a reference situation in which, whatever the BMI, postural and attentional data are similar.

**Table 1:** Summary of the CoP data and RT for the two groups in the two postural conditions (mean ± standard deviation) in simple task (ST) ans dual task (DT) conditions.

		Control			Obese		
		CoP data		RT (ms)	CoP data		RT (ms)
		Speed (mm.s^−1^/BH)	Range (mm/BH)		Speed (mm.s^−1^/BH)	Range (mm.m/BH)	
Seated	ST	1.76±0.22	2.05±0.37		1.9±0.41	3.72±1.21	
	DT	1.74±0.2	1.82±0.33	190.65±32.66	1.82±0.26	3.12±0.86	238.07±52.18
Unipedal	ST	17.77±4.3	25.28±4.45		39.26±17.09	36.94±7.98	
	DT	19.17±4.09	24.73±4.71	205.48±52.98	38.21±14.98	39.8±11.2	317.43±87.46

**Figure 1:**
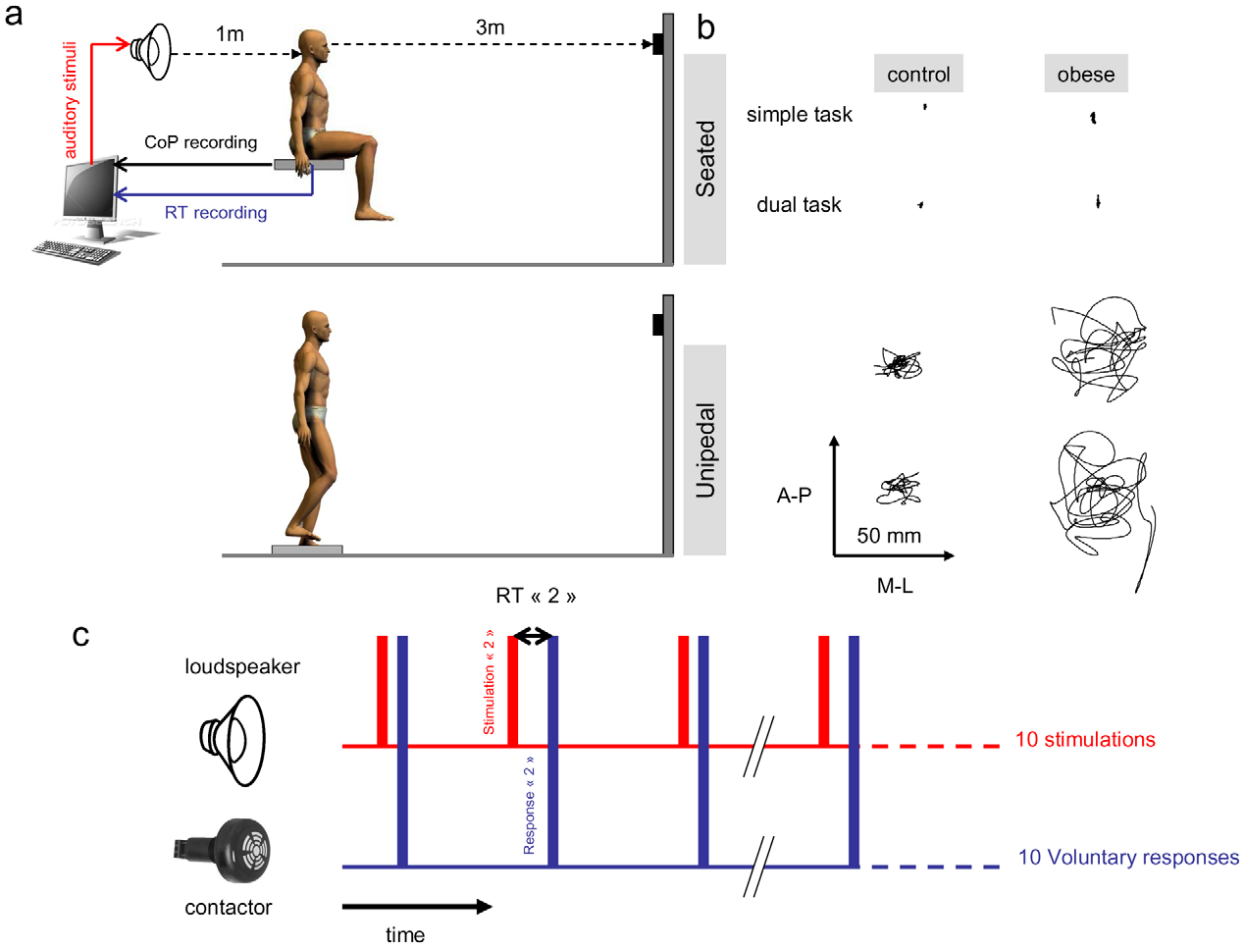
Experimental setup (a). Illustration of CoP displacement on the AP and ML axes in the single and dual-task conditions in the seated and unipedal postures for a non obese and obese patient **(b)**. Auditory stimulus representation and motor response via the contactor. At each stimulus was associated a RT defined as the time difference between the signal peak of stimulation and the motor response **(c)**.

In the unipedal position, the biomechanical configuration (the “centre of mass height/base of support” ratio is clearly higher than the one observed in a seated posture) placed participants in a vulnerable situation in which personal integrity was clearly at risk. As a result, CoP sway (i.e., range and speed) was obviously greater for the controls and obese patients in contrast to the sitting condition. The increase of CoP range reflected the existence of sudden and severe displacements of the CoP towards the limits of the base of support (**figure 1b**). Similarly, the increase of CoP speed reflected the quantity of activity necessary for controlling posture and could be interpreted as a deficit of postural control. These increases were larger for the obese patients than for the control group suggesting that for the control group, postural CoP oscillations were relatively limited and maximal CoP excursions remained at distance of the base of support borderline in comparison to the obese patients. For the obese patients, however, the biomechanical constraints imposed to counteract a same postural disruption and to return to an average CoP position required to generate a muscular torque which magnitude must be higher and/or generated faster than in non-obese subjects [7].

**Figure 2:**
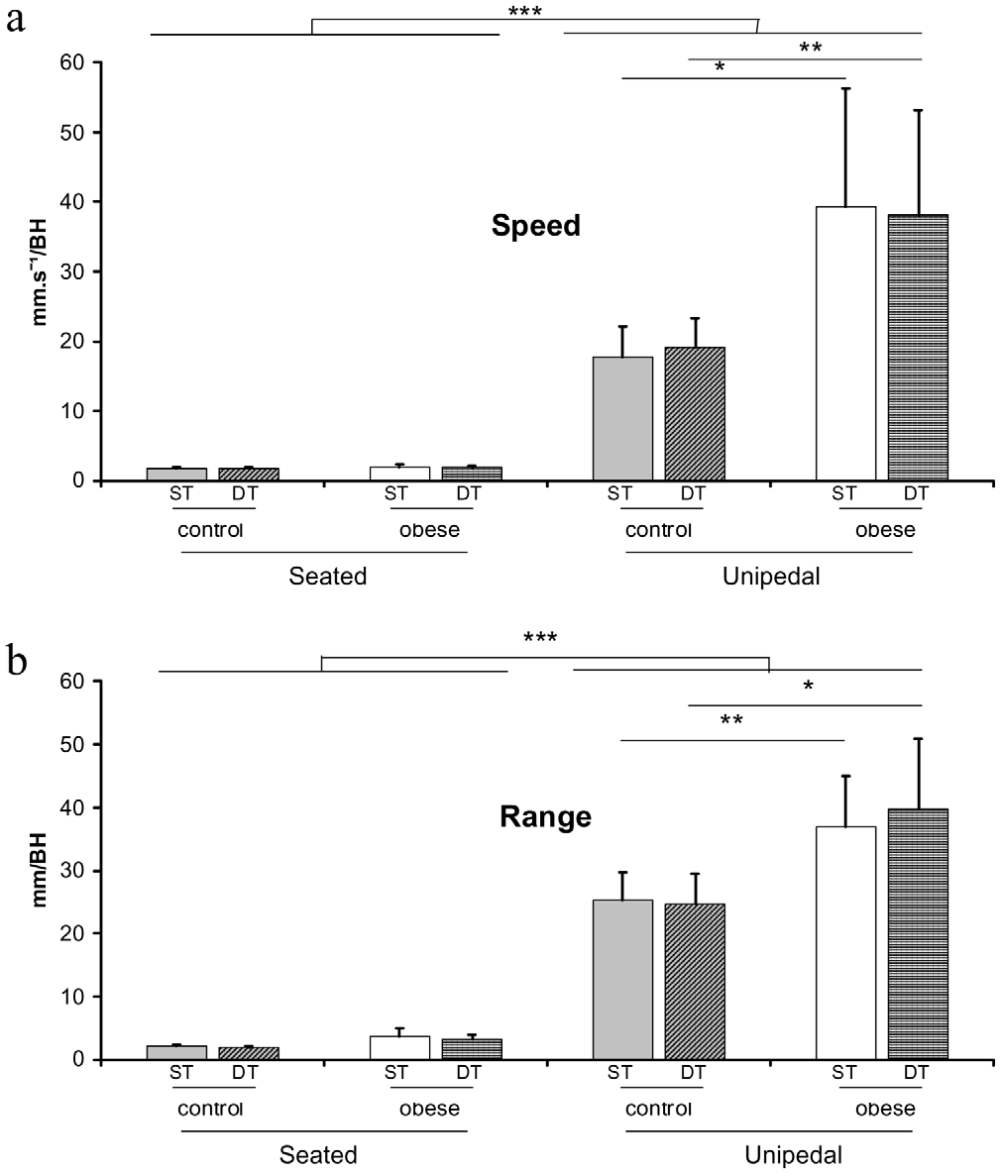
Mean CoP speed (a) and range (b) and standard deviation for the two groups and the two postural conditions in the simple postural task (ST) and dual-task (DT).

**Figure 3:**
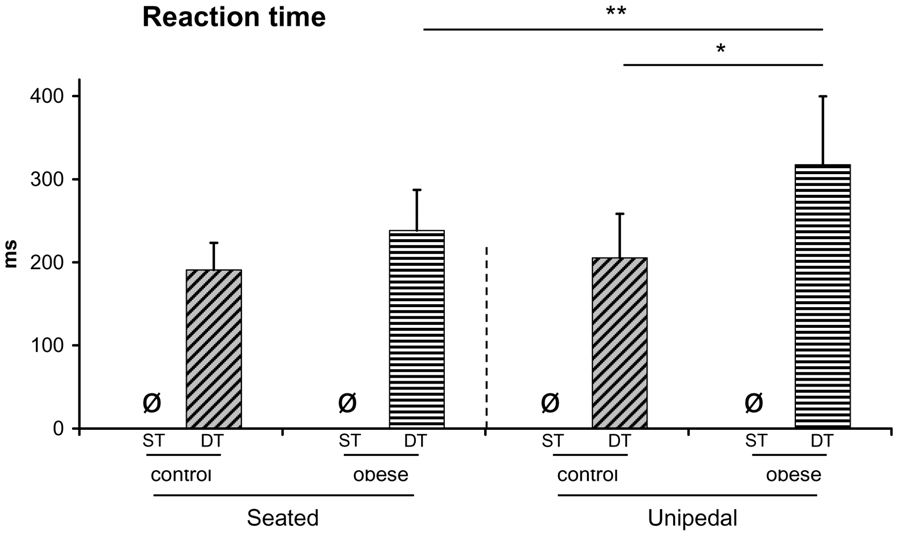
Mean RT and standard deviation for the two groups in the two postural conditions.

More specifically, similar CoP oscillations in the simple and dual-tasks conditions were observed for both groups (**figure 2** **,** **table 1**). However, whereas control participants were able to maintain a similar RT performance whatever the postural complexity in the dual-tasks condition, obese patients exhibited much longer RTs (RT increased by over 33% in average) in the unipedal stance as compared to the baseline sitting condition (**figure 3** **,** **table 1**). In other words, for the healthy participants, maintaining a unipedal posture did not seem to require a greater amount of attentional resources suggesting that the neural processes for preserving this erect posture can be regarded as rather automatic. Conversely, for the obese patients, the increased RT observed in the same postural condition reflected a higher attentional cost for controlling the unipedal stance because controlling this complex posture probably required a supra-spinal process. In other words, when it was necessary to control online their posture (as it was the case in the unipedal condition), obese patients exhibited some difficulties. Similar interpretations have been proposed in studies investigating postural control in elderly people [13], [14], [31].

The exclusive focus of attention on postural stability generally results in larger CoP displacements in a situation in which subjects do not need to focus voluntarily on postural stability [32]. Only in situations in which the additional cognitive task is truly complex, postural sway increases [33]. These observations were generally made while maintaining a bipedal stance. However, this postural task does not seem complex enough to induce a risk of falling. In the present experiment, no instructions were given to participants regarding the priority dedicated to one or the other task. In obese patients, the absence of postural difference between the single- and dual-tasks conditions and the concomitant RT increase during unipedal stance highlighted the priority these obese patients dedicated to postural control. In the unipedal single-task condition, obese patients were already experiencing critical values of CoP displacement. If the obese patients reduced the attention dedicated to the control of posture during the dual-tasks condition, they would probably fall. Thus, the obese patients preferred to decrease their performance in what they considered to be the so-called “secondary” task (increased RT to auditory stimuli), which did not affect their physical integrity. However, in everyday life there are many situations in which we need to manage different attentional tasks together with balance and/or locomotion. The present results clearly suggested that obese patients are not able to easily perform multitasking as healthy adults do.

From a neurophysiological point of view, we hypothesized that skin stretching resulting from obesity may increase the distance between the cutaneous mechanoreceptors and may thus decrease the discrimination threshold of somato-sensory perception. Additionally, a recent study [34] showed that proprioception at knee joint is already altered with this pathology in young obese patients aged 7 to 12. Body schema is built on the basis of multisensory inputs including cutaneous and proprioceptive receptors. With obesity, these receptors may provide altered information to the somato-sensory cortical area altering in turn the obese patients' body schema representation. In addition, it has been shown that the limited physical activity, as generally reported in obese patients, also contributed to the alteration of the body schema [35]–[36]. To develop muscular responses adapted to the postural constraints, the internal model for action must be based on an appropriate body schema [37]. Therefore, we believe that obesity altered the subjects' body schema and internal models necessary for postural control, especially in complex postural tasks.

Plasticity of the neuro-muscular system and appropriate internal models may allow better adapting the neurophysiological constraints to this pathology to answer clinical problems such as balance disorders. Some authors highlighted the benefits of weight loss on postural control improvement [38]–[40]. Associated to weight loss, a rehabilitation program could be optimized by combining physical practice (Strength training, daily living activities, balance training [39]–[40]), motor cognitive training (mentally simulated motor action [41]–[42] or virtual reality [43]) and learning to better use the sensory information available (e.g., visual anchoring [44]). Combining these ways of rehabilitation could act synergistically to improve postural control in obese patients.
